# Post-operative management and follow-up of surgical treatment in the case of rectovaginal and retrocervical endometriosis

**DOI:** 10.1007/s00404-020-05686-0

**Published:** 2020-07-13

**Authors:** Elene Abesadze, Vito Chiantera, Jalid Sehouli, Sylvia Mechsner

**Affiliations:** 1 Department of Gynaecology, Charité - University Clinic, Endometriosis Centre Charité, Campus Virchow Clinic, Berlin, Germany; 2 University of Palermo, Palermo, Italy

**Keywords:** Rectovaginal endometriosis, Retrocervical endometriosis, Pelvic pain, Surgical technique, Recurrence rate, Fertility rate

## Abstract

**Introduction:**

Deep infiltrating endometriosis (DIE) affects between 3.8% and 37% of all endometriosis patients, mostly affecting rectovaginal septum or retrocervical space and characterized by the severe endometriosis-related complaints. Nowadays, generally managed with surgery. However, this is associated with a risk of postoperative complications. To better evaluate intra- and postoperative complications and outcomes for rectovaginal (RVE) and retrocervical endometriosis (RCE), the preoperative management should be accurately described and compared.

**Methodology:**

This is a cohort retrospective study performed at the Endometriosis Centre of Charité-University Clinic, Berlin. 34 patients were investigated in their reproductive age, *n *= 19 with RVE and *n *= 15 RCE, operated between 2011 and 2015. The surgical approach was divergent in both groups. Single laparoscopy was performed in RCE patients (RCEP) and vaginal assisted laparoscopy in RVE patients (RVEP). Long-term postoperative outcome included complications, fertility rate and recurrence rate.

**Results:**

The median follow-up time was three years (y). Symptom-free status was revealed in *n *= 12 RVEP and *n *= 9 RCEP. Postoperatively, endometriosis-related complaints were presented in *n *= 7 RVEP and *n *= 6 RCEP, but with significant pain relief. From n = 8 RVE patients seeking fertility, pregnancy occurred in n = 7 and from n = 9 RCEP pregnancy appeared in *n *= 5 patients in the meantime of 6 months. Postoperative complications were reported in *n *= 1 RVEP with early postoperative bleeding, after ureter leakage and *n *= 1 RCEP with postoperative anastomotic insufficiency. The postoperative recurrence rate was equivalent to zero.

**Conclusion:**

The appropriate surgical approach for each group, preserving anatomy and functionality of the organs, seems to be very essential and efficient.

## Introduction

**Fig. 1:**
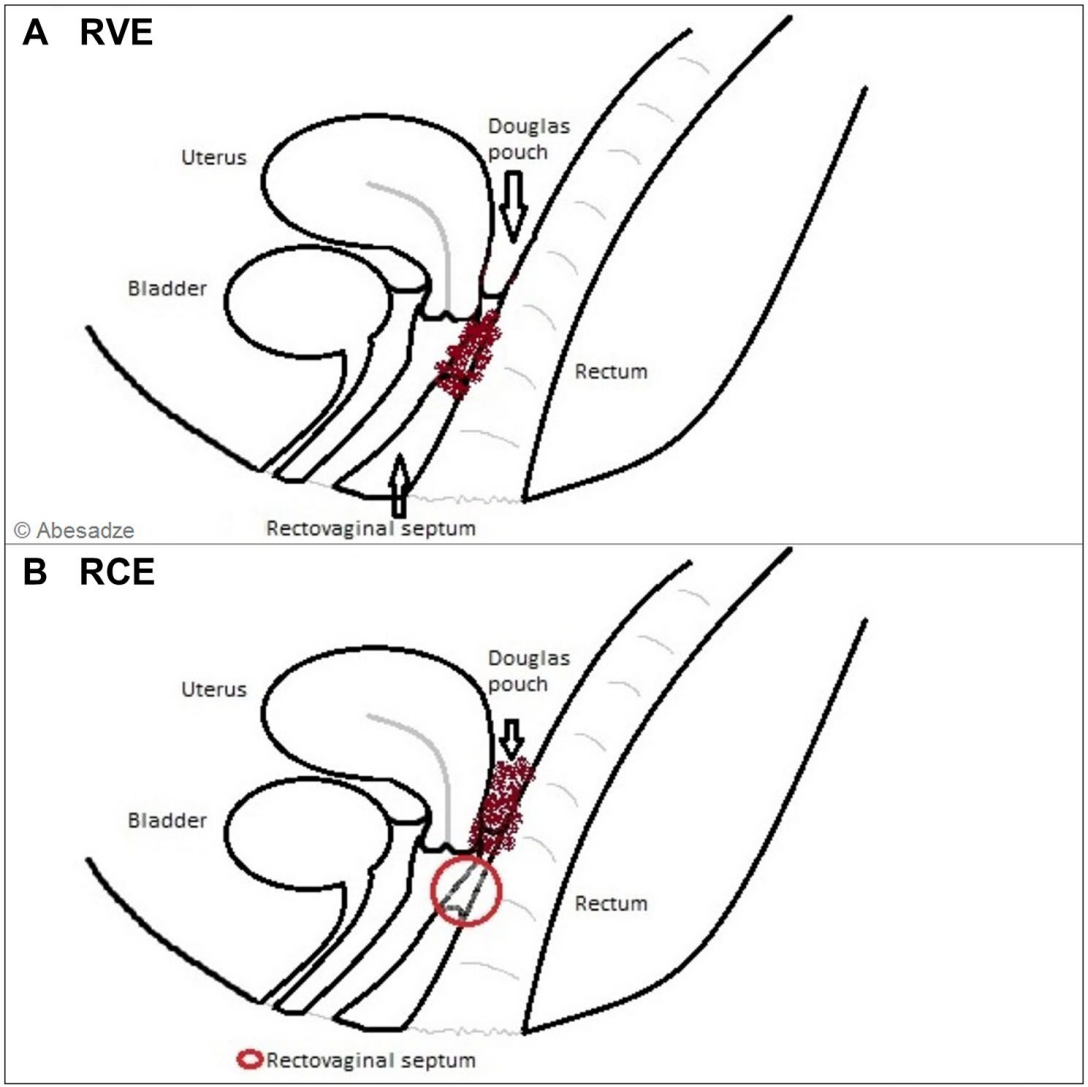
Anatomical terms of location for rectovaginal (**a**) and rectocervical (**b**) endometriosis

Endometriosis (EN) is the most frequent disease of the pelvic cavity in women of reproductive age [1]. Three different clinical presentations of EN lesions (EL) could be considered: peritoneal endometriosis, endometriomas and deep infiltrating endometriosis (DIE) [2]. DIE is the most severe form of lesions, because of infiltration and damage of adjacent organs like the bowel, the bladder and/or the ureter and effects around 10% of all endometriosis patients [3].The most frequent affected area is the pouch of Douglas. Here, two manifestation forms of DIE, the rectovaginal with infiltration of the rectovaginal septum with or without infiltration of the vagina and retrocervical endometriosis [3–5] (Fig. 1) could be distinguished.

The exact definition of the topography of retrocervical and rectovaginal EN is mandatory for the understanding of the disease and for planning the surgical treatment. The term rectovaginal EN (RVE) is often used for both retrocervical EN (RCE) and RVE [4], even though there are actually significant differences between these two terms and the surgical approach.

Moreover, Martin and Batt defined that an infiltration of the retroperitoneal space and posterior vaginal fornix behind or beneath the cervix is typically without rectal involvement, and is more typical for RCE [6] and the involvement of rectum, vagina, rectovaginal pouch and sometimes RV septum is more common RVE [6]. Clarified terminology is a basis for accomplishing the correct preoperative evaluation, informed consent, and intraoperative approach. In particular, the treatment of RCE is less complex than the treatment of RVE [6].

Another essential issue is a proper examination of the patient. Considering that, the complaints like, dysmenorrhea, pelvic pain, dyschezia, dyspareunia, sexual impairment and impaired fertility are similar for both RVE and RCE. Therefore, the right diagnosis is always a challenge. In both cases, it is essential to complete a bimanual examination with a rectovaginal examination to inspect the posterior vaginal fornix, uterosacral ligaments, paracervical tissue, softness of rectal and vaginal wall and finally the infiltration of the rectum. Nevertheless, in some cases, the rectal infiltrations are further away from the anus in the rectosigmoid colon and hard to palpate. Furthermore, vaginal ultrasound is mandatory to confirm involvement of the rectovaginal septum and bowel infiltration [7, 8]; it also a good way to differentiate rectovaginal from retrocervical endometriosis. To complete the preoperative planning, additional diagnostic methods like endosonography, sigmoidoscopy and magnetic resonance imaging (MRI) are necessary.

To date, surgical therapy is the first-rate treatment for symptomatic RCE and RVE as medical therapy is usually only temporarily effective and a complete excision of endometriotic tissues improves the chance to avoid recurrence and obtain a better follow-up [5, 9]. However, surgical treatment is complex and associated with complications. Our target was to evaluate the efficiency of laparoscopically assisted vaginal approach and single laparoscopy in two similar terms anatomically located on the different sites of the rectovaginal area, but also side-by-side. The purpose of this study was to analyze: the probability of minor and major complications pre- and postoperatively (like suture insufficiency), recurrence rate and postoperative outcomes. To our knowledge, there is no current study assuming the coordinated surgical approach for RVE and RCE.

## Materials and methods

This is a single-center study comprising two groups of patients with symptomatic RVE and RCE (Table 1). This study was focused on a particular group of women of reproductive age with current or prospective family planning. The mean age for RVEP was 34 year (± 5.4)) and 31 year (± 4.8) for RCEP(*n *= 17 seeking fertility, of which *n *= 8 RVE and *n *= 9 RCE). We excluded patients who had undergone a hysterectomy because an organ sparing surgery was mandatory. All 34 patients were operated on between 2011 and 2015 at our department of gynecology Charité, Berlin Germany and postoperative follow-up was made from 2012 till 2018. Preoperative risk scores were assigned to patients. The study patients were divided into two groups. The first group comprised 19 women with RVE, *n *= 11 (57%) with and *n *= 8 (43%) without bowel infiltration and the second group presented *n *= 15 patients with RCE, *n *= 10 (66%) with and *n *= 5 (34%) without bowel infiltration. The database was collected from the clinical history of patients. All 34 women were examined and diagnosed in our outpatient clinic. The clinical data were analyzed from the gynecological and digital examination. To evaluate suspected bowel infiltrations, additional diagnostic methods were performed such as endosonography, colonoscopy and MRI. On the day of the visit, each patient took part in a verbal questionnaire based on visual analog scale (VAS) and numerical rating scale (NRS).

**Table 1:** General overview of the preoperative findings, surgical procedures and follow-up in 34 study patients

	RVE (*n *= 19)	RCE (*n *= 15)
Mean time of the follow-up, month (± SD)	3 years (± 1.3)	2 years (± 1.2)
Mean age of the patients, year (± SD)	34(± 5.4)	31(± 4.8)
Preoperative hormonal treatment, *n* (%)	12	9
*Pre-operative infertility rate n* (%)	8 (23, 5%)	9 (26, 4%)
Adenomyosis *n* (%)	14 (41%)	12 (35%)
Previous surgery for endometriosis, *n* (%)	10 (53%)	9 (60%)
Diagnostic laparoscopy with biopsy, *n* (%)	2 (11%)	3 (20%)
Incomplete excision, *n*(%)	4 (21%)	5 (33%)
Recurrence of EL, *n* (%)	4 (21%)	1(6%)
*Procedures*		
The mean time of the operation	4.3 h	3.2 h
Retrocervical EN excision *n* (%)	0	15 (100%)
Rectovaginal EN excision *n* (%)	19 (100%)	0
Vaginal dissection of EN *n* (%)	10 (29%)	0
Cyst excision *n* (%)	6 (31%)	8 (53%)
Hysterectomy *n* (%)	0	0
Partial bowel resection *n* (%)	10 (57%)	10 (66%)
Protective ileostomy	2 (11%)	4 (27%)
Partial bladder resection *n* (%)	4 (21%)	2 (13%)
Neurolysis *n* (%)	14 (74%)	15 (100%)
Ureterolysis *n* (%)	13 (68%)	13 (86%)
Peritonectomy	9 (47%)	12 (80%)
Adnexectomy	3 (16%)	0
Appendectomy	1 (5%)	1 (6%)
Diaphragm resection	0	2 (13%)
Lymphadenectomy	1 (5%)	0
Umbilicus partial resection	1 (5%)	0
*Follow*-*up*		
Symptom free status postoperatively *n* (%)	12 (63%)	9 (60%)
Postoperative pregnancy *n*=	7	5
Recurrence, *n*	0	0
Complication (%)	1 (2, 9%)	1 (2, 9%)

*SD* standard deviation

### Surgery

The surgical tactics were divergent and performed by the same team: including vaginal-combined assisted laparoscopy in case of RVE [10] and single laparoscopy in patients with RCE [9]. The mean time of RVE operations was 43 h and 32 h for RCE. In most of the cases, the disease was complex, involving other organs as well. Intraoperatively the severity and localization of the EN was assessed, using classification of the American society of reproductive medicine (rASRM) and a classification of Deep Infiltrating Endometriosis (ENZIAN score) (Tables 2, 3). The vaginal-combined laparoscopic technique started with the vaginal excision of the infiltrated area which was presented in Possover’s work from 2000 [10]. It was followed by the preparation of the rectovaginal septum. The prepared part of the vagina was shifted on the rectum. The posterior fornix of the vagina was sutured to the posterior cervix. Later proceed with laparoscopical separation of the retroperitoneum along the coccygeal–sacral bone towards transvaginally exposed area, including nerve-sparing and laparoscopic neuro-navigation technique performed by the Possover’s technique as well [11]. In the case of recto-sigmoidal infiltration, the whole conglomerate together with rectal EN was prepared and transected cranially by the laparoscopic stapling device, followed by the suprapubic laparotomy in order to prepare the bowel for anastomosis. In the final step, the transanal stapled anastomosis was completed. We had three cases of RVE, where the EL were not expanded too deep in the vagina, approximately 2 cm in diameter. Here, the lesions were resected with the single laparoscopy as one en bloc specimen, including entire posterior peritoneum of the pelvis, in two cases with and in one without vaginal resection. Here, the vaginal suture was applied laparoscopically as well. This procedure was only part of the complex surgical treatment, including partial bladder resection *n *= 4 (21%, out of *n *= 19 RVEP), ureterolysis *n *= 13 (68%), neurolysis *n *= 14 (74%, in particular thirteen hypogastric nerve and one sacral plexus), partial nerve resection *n *= 1 (5%) including hypogastric nerve, peritonectomy *n *= 9 (47%), unilateral adnexectomy *n *= 3 (16%), lymphadenectomy *n *= 1 because of lymph node extension (not symptomatic) (5%) and partial umbilical resection *n *= 1 (5%) (Table 1).

**Table 2:** Summary of preoperative and intraoperative rASRM classification in patients with RVE and RCE

rASRM staging	RVE	RCE
rASRM I *n *= 4	3	1
rASRM II *n *= 9	7	2
rASRM III *n *= 4	1	3
rASRM IV *n *= 17	8	9

**Table 3:** The intraoperative classification of endometriosis with ENZIAN score for RVEP and RCEP

	ENZIAN 1 < 1 cm			ENZIAN 2 1–3 cm			ENZIAN 3 > 3 m			ENZIAN F				
	A	B	C	A	B	C	A	B	C	FA	FB	FU	FI	FO
RVE	2	1	1	8	1	6	5	1	4	14	4	0	2	3
RCE	2	0	0	0	3	4	5	0	7	12	2	1	2	2
Total *n*=	4	1	1	8	4	10	10	1	11	26	6	1	4	5

In all patients with RCE (*n *= 15), a single laparoscopy was performed. Uterine manipulator was applied to displace the uterus anteriorly and push up the posterior fornix better. The procedure started with adhesiolysis and opening of the retroperitoneal space, presenting the ureter on the both sides in some cases (*n *= 13); followed by the preparation of pararectal spaces, exposing superior hypogastric nerves and pelvic splanchnic nerves. The final step was the peritonectomy of the complete posterior compartment, with the excision of the cervical nodule in one en bloc sample. The RCE surgical procedure was also extended, along with partial bowel resection 10 (66% out of *n *= 15 RCEP), partial bladder resection 2 (13%), neurolysis 15 (100%) including hypogastric nerve, partial nerve resection *n *= 6 (40%) counting five hypogastric nerve and one plexus sacralis, ureterolysis 13 (88%), peritonectomy 12 (80%), appendectomy 1 (6%), diaphragm resection 2 (13%). All specimens were sent for histopathological examination and endometriosis was proven histopathologically. Table 1 demonstrates an overview of the performed surgical procedures (Table 1).

Post-operative follow-up was evaluated during visits in our outpatient clinic and by phone interview. During the interview, patients were asked questions about symptoms related to dysmenorrhea, dyspareunia, dyschezia, recurrence and postoperative pregnancy, rated by the VAS and NRS as well.

### Statistical analysis

Data analysis included age, pre-operative and postoperative complaints, operative procedures, recurrence rate, complications, fertility rate. The mean and standard deviation (SD) for indications and postoperative follow-up were taken. Divergence between pre- and postoperative status was investigated by Graph pad Prism 5 using *p* value test and *t* nonparametric test.

## Results

### Indications

As our study was focused on the women in their reproductive age, with current and prospective family planning, the mean age of the patients with RVE was 34 (± 5.4)) and 31(± 4.8) for RCEP (Table 1). RVE was presented in 55% (19) of the patients and RCE in 45% (15). All 34 patients took non-steroidal anti-inflammatory drugs (NSAID) preoperatively. Previous hormonal therapy had been taken in *n *= 12/19 (63%) RVEP and in *n *= 7/15 (47%) RCEP, including combined oral or vaginal contraceptives and progesterone-only pills. Surgical treatment was recommended in cases of infertility and ongoing symptoms or with a progression of the symptoms while taking hormonal treatment, which was interrupted 2 months prior to surgery. *N *= 19/34 (56%) of the women were operated on one or more times prior to our surgery, recorded in *n *= 10/19 RVEP and in *n *= 9/19 RCEP. Of this group, *n *= 2/9 RVEP and *n *= 3/9 RCEP underwent a single diagnostic laparoscopy. In *n *= 4/10 RVEP and in *n *= 5/9 RCEP, visible EL were partially resected or coagulated, reporting ongoing EM-related complaints. Preoperatively, an incomplete excision of RVE endometriosis was performed in *n *= 4/10 RVEP and an excision of RCE was done in *n *= 1/9 RCEP; accordingly, these cases indicated a recurrence/progression of RVE and RCE EM. Endometriosis-related complaints were similarly recorded in the RVEP and RCEP. Dysmenorrhea was present preoperatively in *n *= 17 (89%) RVEP and in *n *= 12 (80%) RCEP. Cyclic pelvic pain was reported in *n *= 16 (84%) RVEP and *n *= 11 (73%) RCEP, chronic pelvic pain in *n *= 9 (47%) RVEP and in *n *= 9 (60%) RCEP. *N *= 17 (89%) RVEP and *n *= 11 (73%) RCEP suffered from dyspareunia, and *n *= 15 (79%) RVEP and *n *= 7 (47%) RCEP from dyschezia. Dysuria was documented in *n *= 3 (15%) RVEP and in *n *= 3 (20%) RCEP. Impaired fertility was presented in *n *= 8 (42%) RVEP, with *n *= 7 cases of primary sterility and one case of miscarriage in an early phase. From *n *= 9 (60%) RCEP seeking fertility, *n *= 7 cases of primary sterility, one case of secondary sterility and one case of miscarriage were documented.

### Intraoperative status

The intraoperative staging of the EN manifestation is presented in Tables 2 and 3. The vaginal assisted laparoscopy was performed in 16/19 (84%) RVEP. The remaining three patients (16%) with RVE underwent a single laparoscopy, *n *= 2/19 with laparoscopical vaginal dissection and *n *= 1/19 without. All 15 women with RCE received a single laparoscopy. Rectosigmoid resection was performed on 64% (20) of women, including 10/15 RCEP and 10/19 RVEP. The mean distance of the anastomosis above the anus was 8 cm in both groups, with a minimum distance of 6 cm and maximum 10 cm, similar for both groups. All of these 10 RVE patients underwent vaginal dissection as well, applying a suture vaginally in eight cases and laparoscopically in two cases. No vaginal dissection was carried out in case of RCE. In case of very deep rectum resection, there was constructed protective ileostomy in 6/34 (18%) patients, within 4 RCEP and 2 RVEP (Table 1). In most cases, the excision of RV and RC lesions was a consequence of deep infiltrating endometriosis (Tables 2, 3.) and required complex surgery. The classification of the infiltration, width and depth of the infiltration and, location of endometriosis was made by the ENZIAN score and rASRM staging as previously noted (Table 2, 3.). More than 50% of the patients reported rASRM stage IV (Table 2), which also infiltrated nearby organs. RVE was present in the infiltration of rectosigmoid junction (*n *= 10), bladder (*n *= 2), nerves (*n *= 8), appendix (*n *= 1), lymph nodes (*n *= 1), umbilicus (*n *= 1). In patients with RCE, the pouch of Douglas was partly or totally obliterated, involving rectosigmoid junction (*n *= 11), bladder (*n *= 2) fallopian tubes (*n *= 4), ovaries (*n *= 8), nerve fibers (*n *= 8), appendix (*n *= 1), diaphragm (*n *= 2) in the process.

### Early post-operative complications

Our investigation reported no minor postoperative complications, but two cases of major complications in the early post-operative period: one patient with RVE and another with RCE. The first patient presented a significant hemoglobin drop on the second postoperative day. Suspecting the intraabdominal bleeding, computerized tomography (CT) scan was taken and a laparoscopical revision was performed. Intraoperatively, the bleeding source could not be identified, coagel was removed and no evidence of vaginal or bowel suture insufficiency was determined. Afterwards, on the third postoperative day, an increased creatinine value in the intraabdominal drainage was revealed. A CT urography confirmed a urinary tract leakage on the left side handled conservatively with urethral stent inserted under cystoscopy. This patient was discharged from the clinic in subjective well-being.

The second patient with RCE demonstrated anastomotic leakage on postoperative day nine with increased infection parameters and suspicious fluid in the intraabdominal drainage, which was the immediate indication for surgical intervention. Intraoperatively was detected a vaginal fistula, as a result of anastomotic insufficiency with local infection. In terms of already existing urethral stent (applied during first operation), the ureter suture insufficiency was identified. Laparotomy was finished by the closure of the vagina, reanastomoses of the ureter and application of the colostomy (Hartmann situation), which was closed 1 year later.

### Follow-up

Postoperatively, every patient *n *= 16 (47%) with no reproductive concern took supportive hormonal treatment (HT) *n *= 11 RVEP and *n *= 5 RCEP, except one patient who refused to take HT because of nicotine abuse. HT included progestogen-only pills, combined hormonal contraceptives, gonadotropin-releasing hormone agonists and hormonal intrauterine device (Table 4). Patients with subfertility were divided in two groups, *n *= 11 patients with adenomyosis, who took HT: *n *= 7 gonadotrophin-releasing hormone agonists (GnRHa) and *n *= 4 dienogest shortly (3 months) postoperatively in non-stop modus as a therapeutic purpose to improve the chances of upcoming in vitro fertilization (IVF). And second group with *n *= 6 patients, where HT was not required. Totally HT was taken in *n *= 27 (77%) of all the patients, out of them *n *= 12 RVEP and *n *= 9 RCEP (Table 4).

**Table 4:** Postoperative hormonal treatment in patients with and without subfertility

	Dienogest *N*=	COC *N*=	Desogestrel *N*=	Desogestrel /Mirena *N*=	Single GnRHa *N*=	Mirena *N*=	GnRHa followed by Dienogest *N*=
RVEP ∅ subfertility	7	3	0	1	0	1	0
RCEP ∅ subfertility	3	0	0	0	0	0	1
RVEP with subfertility	2	0	0	0	1	0	0
RVEP with subfertility	4	0	1	0	3	0	0

**Fig. 2:**
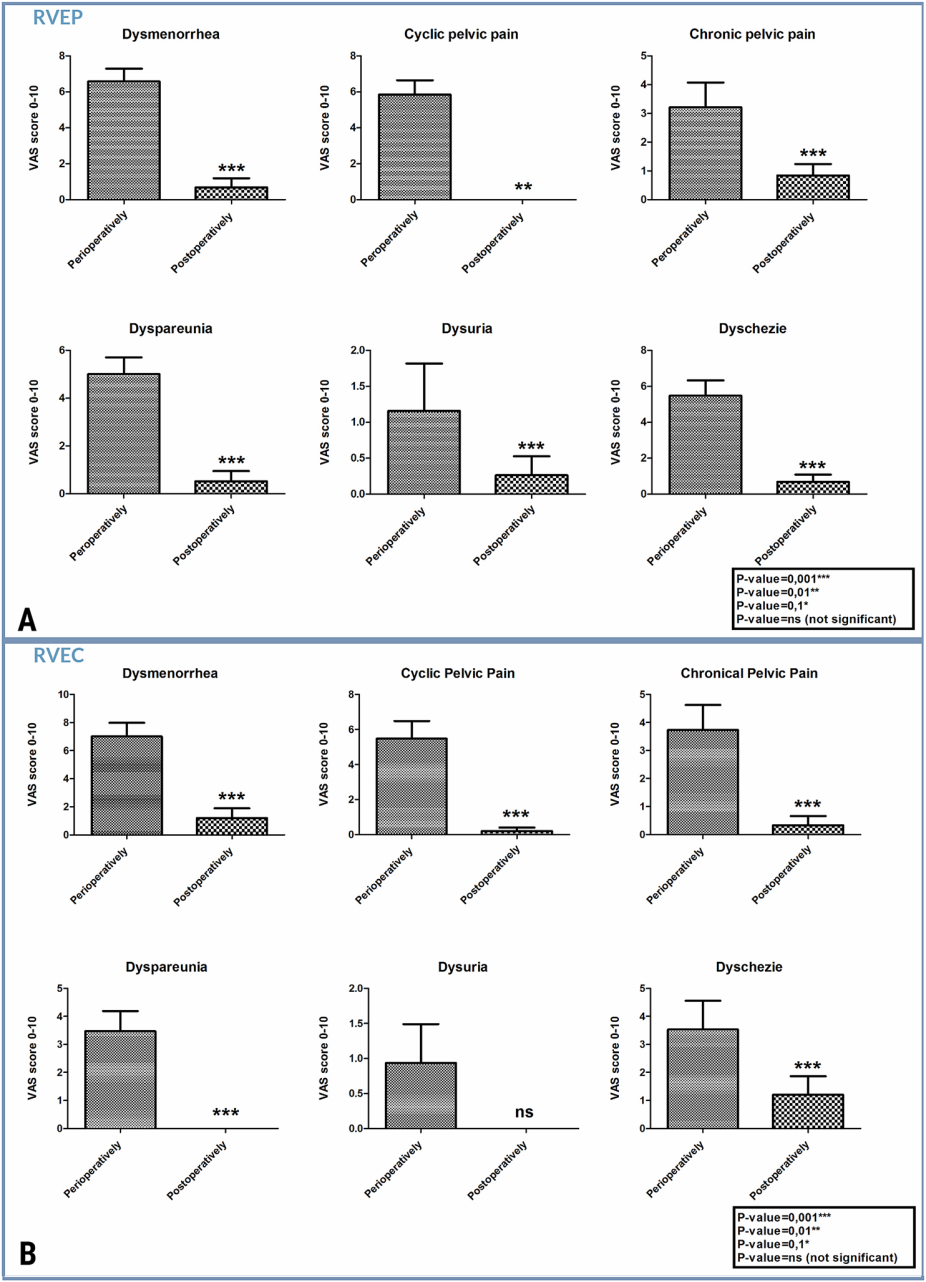
VAS score analyzed statistically by Graph pad Prism 5 using p-value test and t nonparametric test. Graphs present the pre- and postoperative complaint’s considering VAS score, reporting the pain reduction postoperatively in both groups: **a** RVEP and **b** RCEP

In general, postoperative follow-up revealed an apparent reduction of EN-related symptoms. Dysmenorrhea disappeared postoperatively in *n *= 16/17 (94%) RVE and *n *= 9/12 (75%) RCE patients (Fig. 2). Here, we have to mention that one RVEP with remaining postoperative dysmenorrhea had no postoperative HT, as the patient was seeking fertility. Three RCEPs with postoperative dysmenorrhea did not undergo postoperative HT as well, as one was seeking fertility and rest two patients declined recommended postoperative HT. In fact, this group of patients with remaining dysmenorrhea reported decrease of pain intensity according to the VAS score (from 0 to 10 scale system of the pain level), for one RVEP from level 5 to level 4 and for RCEPs from mean level of 8 to mean level of 6. As for the rest *n *= 16 RVEP and *n *= 9 RCEP without postoperative dysmenorrhea, every patient from this group underwent postoperative supportive HT in non-stop modus, except *n *= 3 RVEPs those seeking fertility. However, terminating menstrual bleeding in this group could indicate the absence of postoperative dysmenorrhea. In general, the mean intensity of postoperative persisting symptoms was significantly decreased from level 7 to level 4 (Fig. 2). The postoperative database reported only one case/11 of cyclic pelvic pain in RCEP. Postoperative chronic pelvic pain (CPP) was reported only in *n *= 4/9 RVEP, out of them *n *= 3 took postoperative HT. CPP was reported in one/9 RCEP; this patient was under HT postoperatively as well. There were representative changes in patients with dyspareunia 28 (91%), including *n *= 17 RVEP and *n *= 11 RCEP. This group postoperatively reported only *n *= 2/17 (6%) cases of dyspareunia, significantly only in RVEP. Dyschezia 22 (64%) was one of the main complaints in RVEP 15 (79%) and RCEP 7 (47%), postoperatively reporting only in *n *= 3/15 (21%) RVEP and *n *= 4/7 (57%) in RCEP. From six (18%) women preoperatively suffering from painful urination, *n *= 3 (15%) RVEP and *n *= 3 (20%) RCEP, only *n *= 1/3 RVEP reported dysuria (Fig. 2).

### Fertility rate

**Fig. 3:**
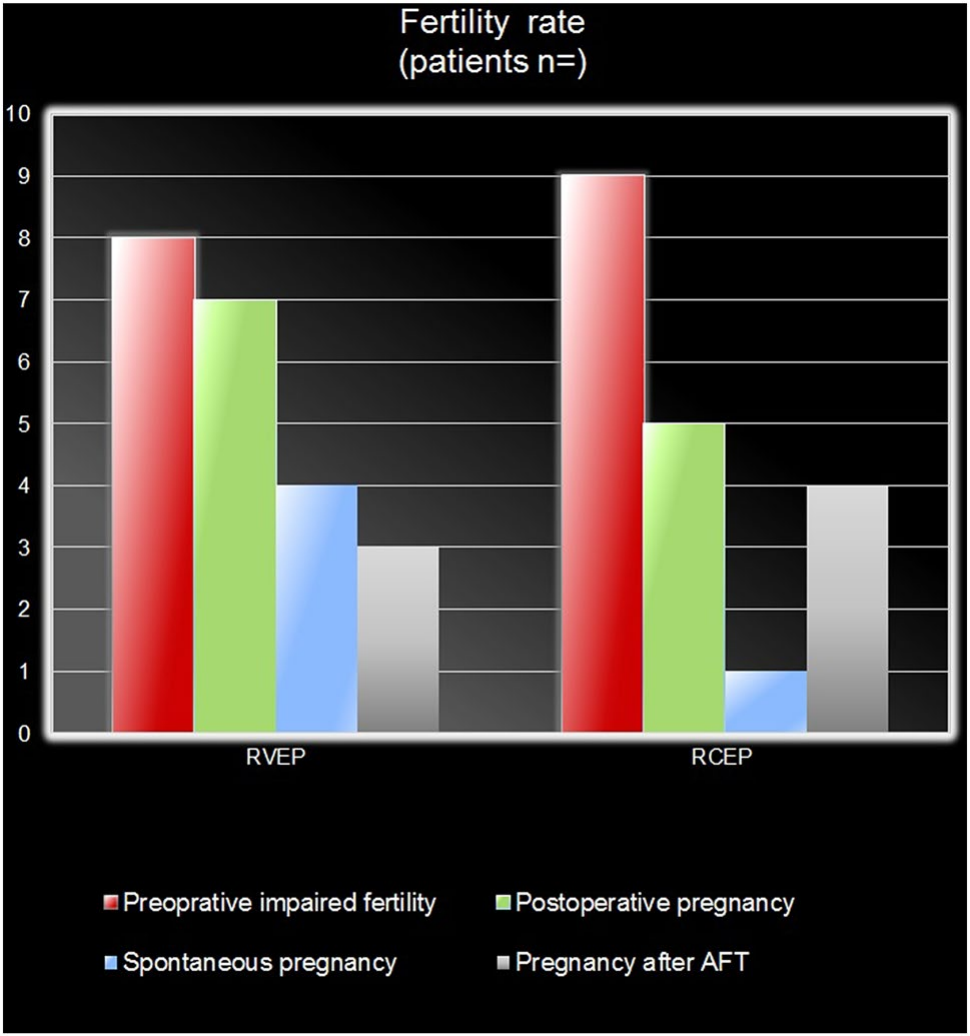
Fertility rate in RVEP and RCEP pre- and postoperatively

The analysis demonstrated an increase in postoperative fertility rate. Preoperatively, *n *= 17 (50%) women suffered from impaired fertility, *n *= 8/19 (42%) RVEP and *n *= 9/15 (60%) RCEP. The median duration of infertility for RVEP was 2 years and 3 years for RCEP. *N *= 14/17 (82%) had a history of primary sterility, *n *= 7 with RVE and *n *= 7 with RCE. The remaining *n *= 3/17 patients suffered from secondary sterility, of which *n *= 2/3 reported a miscarriage in the early weeks of pregnancy, one RVEP and one RCEP, and *n *= 1/3 RCEP reported secondary sterility after childbirth. Postoperatively, *n *= 12/17 (71%) women got pregnant in a mean time of 6 months (Fig. 3), *n *= 7/10 RVEP and *n *= 5/10 RCEP. In *n *= 5/11 pregnancy developed spontaneously, in *n *= 4/5 RVEP and *n *= 1/5 RCEP. Another six patients achieved pregnancy after artificial reproduction treatment (ART) including *n *= 3/5 RVEP and *n *= 4/5 RCEP. In these cases, pregnancy was followed by the delivery of healthy newborns.

**Fig. 4:**
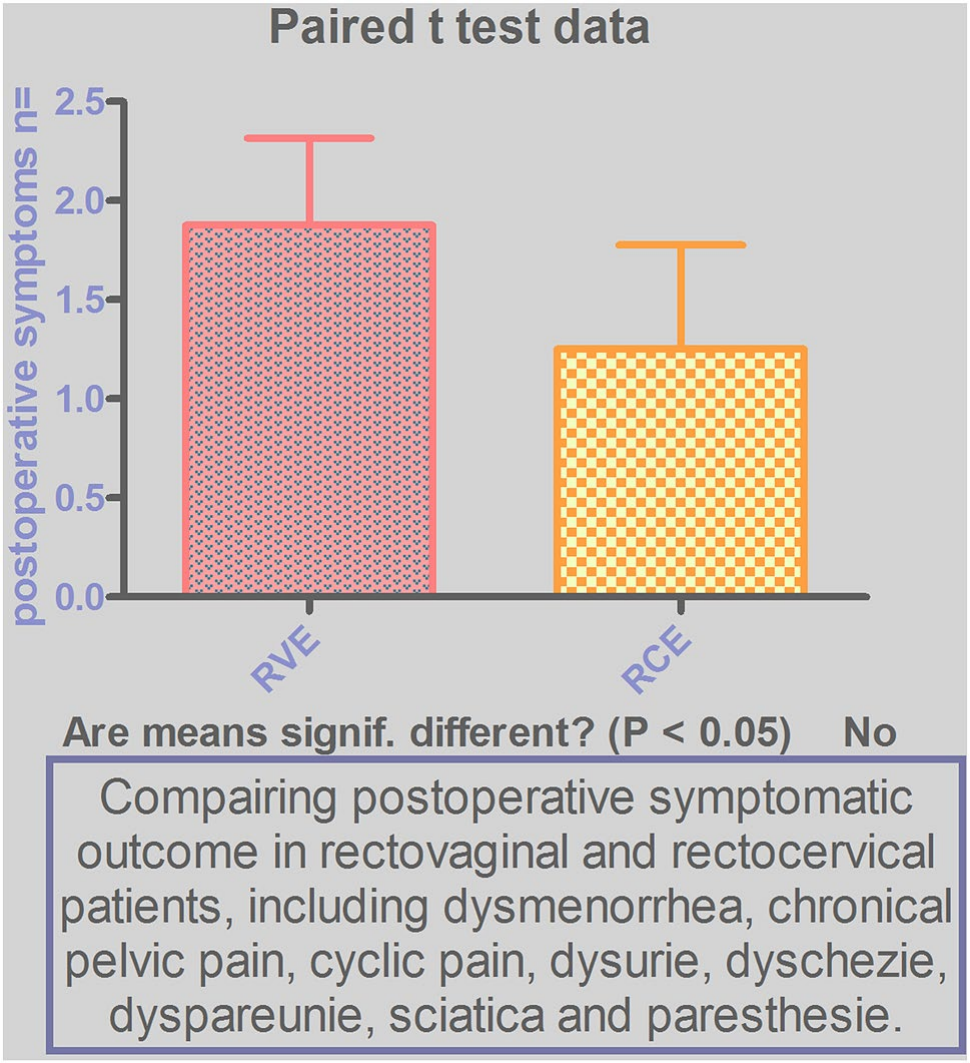
The analysis of postoperative results, presenting no significant difference between RCE and RVE

A paired T-test demonstrated no statistically significant difference in pain relief, postoperative complications or organ dysfunction, fertility rate and recurrence rate between RVE and RCE. (Fig. 4).

## Discussion

In this study group, with a mean age of 32 years, the women are of reproductive age and need an accurate surgical treatment with organ sparing techniques, i.e., the most possible radical excision of the lesions while preserving the functionality of the organs. This was very challenging. Women who had a hysterectomy were excluded from this study.

Furthermore, the larger the EN nodule, the higher the risk of complication [12]. On another hand, the danger of recurrence is higher in cases of incomplete excision of EL [9]. Moreover, a delay in first-line surgical treatment may lead to irreparable consequences in young women seeking fertility [13] such as the future evolution of the disease, reduced ovarian reserve, severity of symptoms and radical surgery (hysterectomy). The complete excision is essential to improve the fertility rate in these women [13–15].

Another relevant topic is achieving a “correct” preoperative diagnosis of the endometriosis. The exact detection of the EM nodules is of utmost importance in managing patients with RVE and RCE, as these patients are suffering from extensive pain for many years before they receive suitable treatment [17]. Differentiating between rectovaginal endometriosis and retrocervical endometriosis is vital [6]. During ordinary gynecological examination, the rectosigmoid segment has to be palpated [10] to determine the location and then extension of endometriotic lesions. Moreover, the posterior vaginal fornix should be inspected with a speculum, to exclude EM infiltration in vagina, and in terms of infiltration analyze the area of involvement. Several studies highlight the importance of the transvaginal sonography (TVS) as a non-invasive tool for diagnosis of DIE preoperatively [16, 17]. Visualization of sonoanatomical changes in the pelvis is essential [7, 8]. Demonstration of endometriotic involvement/nodules in the recto-vaginal septum represents RVE, and the identification of hypoechogenic irregular structures (EN infiltration) on the cervix involving the upper part of the posterior vaginal fornix determines RCE. Therefore, combining bimanual examination with TVS helps to understand the true localization of DIE. Endosonography and MRI provide further opportunity to expose EN localization [18–20]. In our patients, preoperative rectal examination identified 20 cases with bowel infiltration, which were then verified intraoperatively in the estimated extension.

Generally, RVE and not RCE is believed to present with rectal wall infiltration [6, 21], but our study also reported ten (66%) cases of RCE with rectal lesions, correlated and reported with ENZIAN score and rASRM classification (see Tables 2, 3). Therefore, a clear definition for RVE can be considered as the involvement of the vagina and rectovaginal septum, independent of the bowel infiltration. However, the question is which surgical technique is most optimal for RVE and RCE?

This study involves patients with very complex cases, affecting adjacent organs (bladder, intestines, nerve fibers, lymph nodes, and peritoneum) (Tables 2, 3). In these circumstances, the surgical technique is preoperatively unpredictable, and it is more challenging than usual. In the case of RCE without vaginal wall infiltration, it was reasonable to adopt a single laparoscopy. Moreover, applying a uterine manipulator was better highlighting anatomy of the posterior fornix and preventing excessive vaginal dissection. This method avoids possible damage of cervix and therefore improves the chance of successful pregnancy. RVE presented with different anatomical dissemination of endometrial lesions, including rectovaginal septum and vagina itself. Therefore, for RVE treatment was performed vaginal assisted laparoscopy to access the vaginal wall lesions easily, open the rectovaginal septum [10], dissect the infiltration completely and finally reconstruct the wall with a intravaginal suture. Previously, several groups avoided to resect presented vaginal nodules, concerning that excision would make more harm than EL itself [22]. This study presented 28 (91%) of women, RVE (*n *= 17) and RCE (*n *= 11), preoperatively suffering from dyspareunia, but postoperatively identifying only in two (6%) RVEP. Few studies have shown a huge impact of vaginal resection on the improvement in sexual activity postoperatively, reporting more than 80% women with a significant decrease in the presence and severity of dyspareunia [9, 23, 24].

Although some authors see vaginal openings as a main risk factor for complications [12, 25], this method might be of lower risk for patients with a partial bowel resection. As with applying two sutures at the same time on the intraabdominal side, an intestinal and vaginal suture might increase the chances of complications [26], like suture insufficiency, leakages or fistulas [27, 28]. Therefore, applying the suture intravaginally might be a factor for decreasing the friction between two sutures by having them on separate sides. In this study, from the 10/17 RVEP with partial bowel resection, only one case reported early post-operative complication, but without any evidence of vaginal or bowel suture insufficiency, intraoperatively demonstrating an unclear abdominal bleeding and later ureter leakage. Another case of RCE demonstrated a vaginal fistula in the early postoperative period because of anastomotic insufficiency, although no vaginal dissection was performed. The complete dissection of rectovaginal and retrocervical EL, including bowel resection and anastomosis, may appear too radical and tricky, resulting in high risk of postoperative complications [29] as various studies demonstrated postoperative complications rated from 10 to 18%, including anastomosis insufficiency, anastomotic stricture, urinary dysfunction and massive bleeding in patients after vaginal and partial bowel resection [30–33]. In this study, the bowel infiltration was presented from 6 cm till 10 cm above the anus. It is a very low anatomical localization for the bowel resection. Consequently, applying the protective ileostomy (PI) in patients with deep rectal bowel resection could have minimized the chances of these complications and led to the better recovery. Several studies question efficiency of temporal PI in EN patients with rectal resection [34, 35], although a lot of experienced gynecologists demonstrated improved postoperative outcome after applying PI [34, 36–38]. In this study, no patient with ultralow rectal resection and temporal PI reported any postoperative anastomosis-related complications. Nevertheless, the removal of all endometrial nodules carrying out the nerve structures could lead to the improvement of EN-related symptoms and reduced recurrence rate as well. These are important factors for pain generation in these patients [23, 39–41]. Moreover, endometriotic lesions can create their own autonomic and sensory innervation [41, 42] which can, therefore, be associated with hyperalgesia.

This presented approach to this disease seems to be effective, as the overall results of this study show a significant improvement in pain, fertility rate, quality of life, functionality of bladder and bowel postoperatively (Fig. 2), low rate of complications and recurrence rate (Table 1). 62% (*n *= 21) of the women reported symptom-free status, *n *= 12/19 with RVE and *n *= 9/15 RCE. It must be noted that 74% (*n *= 25/34) of these women took supportive hormonal treatment postoperatively, to avoid recurrent disease and to treat persisting dysmenorrhea (in uterus sparing management). It is an interesting finding, as the preoperative hormonal treatment was not efficient for these same patients. In this study, *n *= 19/34 (56%) women received hormonal treatment prior to surgery and reported insufficiency of the HT whilst 12/19 RVEP (63%) and *n *= 8/19 RCEP (42%) had ongoing severe EN-related complaints. In the case of hormonal insufficiency, the indication of the surgical approach appears to be more effective and beneficial for the treatment of RCE and RVE [5, 9, 10, 43]. Moreover, surgery allows us to remove altered area and additional supportive HT terminates the further expansion of the endometriosis. Accordingly, this leads to an efficient postoperative outcome and improved quality of life. Furthermore, *n *= 11 women suffering from infertility took HT postoperatively in nonstop modus for a period of 3 months, including GnRHa (*n *= 2 RVEP; *n *= 5 RCEP) and dienogest (*n *= 2 RVEP; *n *= 2 RCEP), as for the period of wound healing after the excessive surgery and for the therapeutic purpose before IVF in order to achieve successful pregnancy [44]. The follow-up revealed *n *= 6/11 (*n *= 3 RVEP; *n *= 3 RCEP) postoperative successful pregnancies in these women. Although the remaining *n *= 13 (38%) of the women demonstrated postoperative EN-related complaints, the study reported a significant decrease in severity of symptoms and in the recurrence rate in this group (Fig. 2). *N *= 8 of this group took supportive hormonal treatment postoperatively, instead they had symptoms like CPP, dyspareunia, dyschezia, sciatica and paresthesia, and intensity of the symptoms was decreased from preoperative mean level of 8 (according to VAS score scale) to level 4 postoperatively. Another *n *= 5 patients from this group did not have HT when trying to conceive, their symptoms were dysmenorrhea, cyclic pelvic pain, dyschezia, but also with significant pain relief from preoperative mean level of 8 to level 5. Finally, postoperative statistical analysis reported no significant difference in postoperative outcome, between RCEPs and RVEPs (Fig. 4).

This study, in agreement with other studies, confirmed that the resection of the DIE in cases of RVE and RVE can be efficient in terms of successful pregnancy [13–15, 45]. Postoperatively, *n *= 12 (71%) patients seeking fertility achieved pregnancy in a mean time of 6 months, of which *n *= 7 with RVE and *n *= 5 with RCE (Fig. 3). Interestingly, 50% of conceptions happened spontaneously.

In conclusion, our aim was to demonstrate the importance of planning and performing surgery, respectively, to rectovaginal and retrocervical endometriosis, as well as how the preoperative indication can have a huge impact on the postoperative follow-up and complication rate.

## Abbreviations

ART: Artificial reproduction treatment
CT: Computerized tomography
CPP: Chronic pelvic pain
DIE: Deep infiltrating endometriosis
EN: Endometriosis
EL: Endometriosis lesions
ENZIAN: Classification of deep infiltrating endometriosis
GnRHa: Gonadotropin-releasing hormone agonists
HT: Hormonal treatment
IVF: In vitro fertilization
MRI: Magnetic resonance imaging
NRS: Numerical rating scale
NSAID: Non-steroidal anti-inflammatory drugs
PI: Protective ileostomy
rASRM: Revised classification of Endometriosis by American society of reproductive medicine
RCE: Retrocervical endometriosis
RCEP: Retrocervical endometriosis patients
RVE: Rectovaginal endometriosis
RVEP: Rectovaginal endometriosis patients
SD: Standard deviation
TVS: Transvaginal sonography
VAS: Visual analog scale
Y: Years
